# Increased Peripheral Proinflammatory T Helper Subsets Contribute to Cardiovascular Complications in Diabetic Patients

**DOI:** 10.1155/2014/596967

**Published:** 2014-04-03

**Authors:** Ru-xing Zhao, Wen-juan Li, Yi-ran Lu, Jun Qin, Chuan-long Wu, Meng Tian, Tian-yi He, Shou-nan Yi, Dong-qi Tang, Lei Sun, Li Chen

**Affiliations:** ^1^Department of Endocrinology, Qilu Hospital of Shandong University, Jinan 250012, China; ^2^Institute of Endocrinology and Metabolism, Shandong University, Jinan 250012, China; ^3^Centre for Transplant and Renal Research, Westmead Millennium Institute, The University of Sydney at Westmead Hospital, Westmead, NSW, Australia; ^4^Key Laboratory of Cardiovascular Remodeling and Function Research, Research Center for Cell Therapy, Qilu Hospital of Shandong University, Jinan 250000, China

## Abstract

*Background*. Coronary atherosclerotic heart disease (CHD) is one of the major concerns in type 2 diabetes (T2D). The systemic chronic inflammation has been postulated to bridge the increased risk of cardiovascular disease and T2D. We formulated that increased peripheral proinflammatory T helper subsets contributed to the development of cardiovascular complications in diabetic patients. *Methods*. The frequencies of peripheral total CD4+ T helper cells, proinflammatory Th1, Th17, and Th22 subsets were determined by flow cytometry in diabetic patients with or without CHD (*n* = 42 and 67, resp.). *Results*. Both peripheral frequencies and total numbers of Th1, Th17, and Th22 cells were further increased in diabetic patients with CHD. Logistic regression and categorical cross-table analysis further confirmed that increased proinflammatory Th subsets, especially Th22, were independent risk factors of cardiovascular complication in diabetes. Elevated Th subsets also correlated with increased CRP levels and the atherogenic index of plasma. Moreover, Th1 frequency and Th22 numbers demonstrated remarkable potential in predicting CHD in diabetes. *Conclusions*. Increased peripheral proinflammatory T helper subsets act in concert and contribute to the increased prevalence of diabetic cardiovasculopathy. The recently identified Th22 cells might play an independent role in CHD and represent a novel proxy for cardiovascular risks in diabetes.

## 1. Introduction

The systemic chronic inflammation has long been postulated to be the central link between T2D and its associated complications [1]. Among these onerous complications, coronary atherosclerotic heart disease (CHD) is one of the most perishing concerns characterized by its high prevalence and overwhelming life threats [2]. In line with the chronic inflammation in T2D, it is also well established that inflammation plays a pivotal role in the development of atherosclerosis and CHD [3]. This implies that the systemic inflammation in diabetes may also exert impacts on atherosclerosis and CHD and explains the critically high risk of CHD in diabetes patients.

In the past few years, T lymphocytes, especially CD4+ T helper (Th) cells, were documented as dominant participants in the systemic chronic inflammation [4]. Particularly, Th cells infiltrate into fat and peripheral organs in diabetes prior to the accumulation and activation of macrophages, which were considered as the major mediator and effector in chronic inflammation [5]. Furthermore, an imbalanced Th population, characterized by an increase of proinflammatory Th subsets including Th1, Th17 and/or a decrease of regulatory T cells, was identified as the direct trigger of chronic inflammatory diseases including diabetes and CHD in animal models [6]. We have also testified that elevated proinflammatory Th subsets in human, including the recently identified Th22 cells, may participate in the development of T2D [7]. Parallel with these, accumulating evidences have also emerged supporting the potential role of proinflammatory Th subsets in the development of atherosclerosis and CHD in human [8–11]. In light of previous research, we speculated that the elevated Th subsets would contribute largely to the development of CHD in diabetes patients.

Although the plausible links between proinflammatory Th1 or Th17 cells and CHD have been suggested since their elevated frequencies were frequently reported in CHD by previous publications [8–11], there is still no systemic clinical design or analysis verifying the independent association of specific Th subsets with CHD, especially in diabetic populations. And the role of the newly identified Th22 cells remained unexplored either. In this case-controlled study we investigated comprehensively the independent contributions of specific Th subsets including total CD4+ T lymphocytes, Th1 cells, Th17 cells and Th22 cells to the prevalence of CHD in a diabetic population. And their correlations with plasma inflammatory marker C-reactive protein (CRP) and atherogenic index (AIP) were discussed to evaluate the potential predictive values of specific Th subsets in the incidence of CHD in T2D.

## 2. Methods

### 2.1. Subjects

Subjects were randomly selected from clinically definite T2D inpatients in Department of Endocrinology, Qilu Hospital, between April 2012 and March 2013. A total number of 109 patients including 42 patients complicated with CHD were enrolled to guarantee the sensitivity of our study. A detailed clinical record was kept for each subject including history of disease and physical and laboratory examinations. Exclusion criteria included unclear diagnosis of CHD or any clues of autoimmune disorders, recent infections, tumors, fever of any causes, and significantly elevated erythrocyte sedimentation rate. None of the donors received immunosuppressive or immunomodulatory drugs for at least 3 months when sampling. Our research was carried out in accordance with the Declaration of Helsinki (2008) of the World Medical Association. The study has been approved by the Medical Ethical Committee of Qilu Hospital, Shandong University. Each donor signed a consent document. Baseline clinical parameters were summarized in Table 1. This study was conducted in a blinded manner.

### 2.2. Sample Preparation

Samples were prepared as previously reported [7]. Generally, fasting peripheral blood samples were obtained from each donor at 7 to 8 a.m. All samples were operated within 2 hours after collection. 400 *μ*L of the heparinized peripheral whole blood was diluted with an equal volume of RPMI1640 medium containing 50 ng/mL of phorbol myristate acetate (PMA), 2 *μ*g/mL of ionomycin, and 3.4 *μ*g/mL Monensin (Sigma, Saint Louis, USA) and then cultured for 4.5 h at 37°C, 5% CO_2_. PMA and ionomycin served synergically as T cell activators through non-antigen-specific activation of PKC and rising of intracellular level of Ca2+ mimicking signals induced by the T cell receptor complex. Monensin, a protein transport inhibitor, led to accumulation of cytokines intracellularly, facilitating detection by flow cytometric analysis subsequently.

### 2.3. Cell Staining and Flow Cytometric Analysis

Frequencies of Th subsets were determined according to their specific cytokine patterns as previously described. After incubation, 100 *μ*L of blood was stained with PerCP-Cy5.5 conjugated anti-human CD4 monoclonal antibodies (clone: OKT4, Cat: 85-45-0048-42) at room temperature for 20 min. The cells were then treated with equal volume of Fix-perm reagent A for 15 min and then washed with precooled washing buffer. Subsequently, Fix-perm reagent B was added in further permeabilization for intracellular staining and lysis of erythrocytes. The sample was incubated for 20 min together with PE-conjugated anti-IFN-*γ* monoclonal antibodies (clone: 4S.B3, Cat: 85-11-7319-82), FITC-conjugated anti-IL-17A monoclonal antibodies (clone: eBio64D, Cat: 85-11-7179-42), and eFluro680-conjugated anti-IL22 monoclonal antibodies (clone: 22URTI, Cat: 85-50-7229-42). Isotope controls were used for each staining procedure as negative controls and for fluorescence compensation. All the antibodies were from eBioscience, San Diego, CA, USA. And Fix-perm reagents were from Invitrogen (CA, USA). All samples were washed and then collected by BD AccuriC6 Flow Cytometer. Data were analyzed by FlowJo 7.6. Gating strategy and representative dot plots could be referred to as previously reported [7]. Absolute numbers of specific Th subsets in each subject were calculated from lymphocyte counts, frequencies of total Th cells, and corresponding Th subsets.

### 2.4. Determination of High Sensitive CRP and Atherogenic Index of Plasma

The plasma levels of CRP were determined using a latex-enhanced immunoturbidimetric assay by Roche Cobas Integra 800 full-automated analyzer (Roche Diagnostics). The intra-assay and inter-assay coefficients of variation were less than 5% and 10%, respectively. The lower detection limit was 0.1 mg/L.

The lipid profiles were determined and regularly reported by Central Clinical Laboratory of Qilu Hospital. The atherogenic index of plasma (AIP) was calculated as follows: AIP = (TC-HDL-C)/HDL-C.

### 2.5. Statistical Analysis

All data were given as the mean ± SD or median (range) according to data distribution. Differences of parameters between T2D patients with or without CHD were determined by Student's *t*-test unless the data were apparently of skewed distribution. The Pearson or Spearman correlation test was used for correlation analysis depending on data distribution. Partial correlation test was performed to rule out confounding factors. Logistic regressions and Chi-square test (or Fisher's exact test) were conducted to verify the independent contribution of specific Th subsets to CHD onsets and corresponding values of odds ratios (ORs), and 95% confident intervals (CIs) and *P* were given for each model. All tests were performed and figures were generated by SPSS 18.0 or GraphPad Prism 5.0 system. *P* value less than 0.05 was considered statistically significant.

## 3. Results

### 3.1. Demographic Characteristics of Subjects

Significant differences were found in levels of LDL-L, total cholesterol, CRP, peripheral blood lymphocyte counts, and the logarithmical values of homeostasis model assessment of insulin resistance (Ln(HOMA-IR)). Differences in age, sex, disease durations of diabetes, CD4+ T cell frequencies, and so forth might show a remarkable tendency in increasing CHD prevalence but did not show statistical significance in our study. Consistent with previous publications, these data indicated that dyslipidemia, inflammation, and insulin resistance were determinants of the development of atherosclerosis and CHD. The detailed parameters are summarized in Table 1.

### 3.2. Elevations of Peripheral Th22 and Th17 Cells Were More Remarkable in Diabetic Patients Complicated with CHD

**Figure 1:**
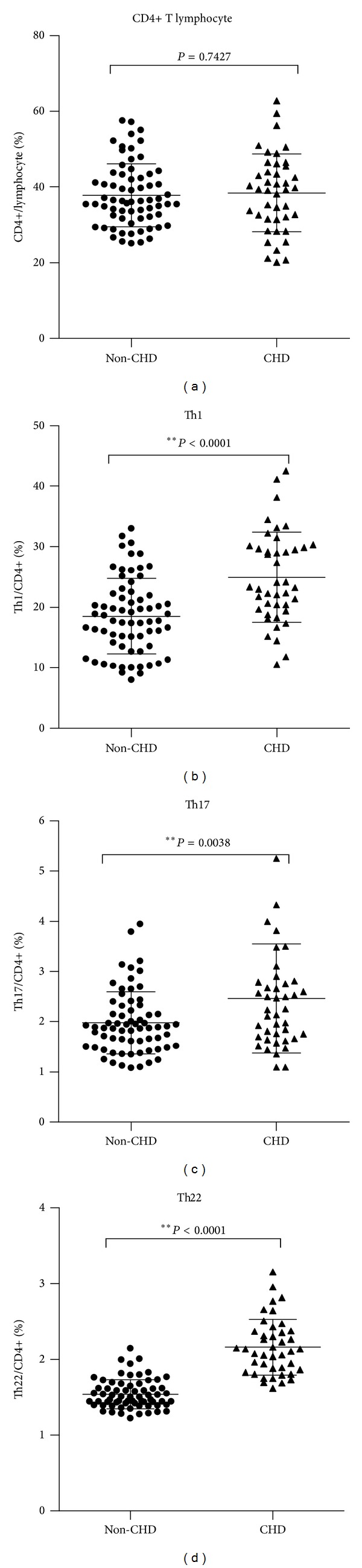
Peripheral frequencies of Th22, Th1, and Th17 Cells were significantly higher in diabetic patients with CHD than those without CHD. Circulating percentages of CD4+ T cells in total lymphocytes as well as Th1, Th17, and Th22 cells in total CD4+ T cells from diabetic patients with (CHD group) or without coronary heart disease (non-CHD group). (a) There was no significant difference in percentages of CD4+ T cells between non-CHD and CHD group (*P* = 0.7427). (b) Significant increases in Th1 were observed in CHD group (24.9 ± 57.46%) compared to non-CHD group (18.53 ± 6.22%) (*P < 0.0001). (c) Significantly elevated percentages of Th17 cells were also found in CHD group (2.46 ± 1.08% versus 1.98 ± 0.62%) (*P = 0.0038). (d) Elevations in Th22 cells in CHD were most remarkable (2.16 ± 0.37% versus 1.54 ± 0.19%) (*P < 0.0001). Bars represent SEM.

We have first reported an increase in peripheral Th22 together with Th1 and Th17 in T2D patients. By further observation, diabetic patients with CHD appeared to show even impressively higher Th cells frequencies. We thus testified this hypothesis in our case-controlled study. As shown in Figure 1, the frequencies of Th22 (CD4+ IFN-*γ*− IL-17− IL-22+), Th17 (CD4+ IFN-*γ*− IL-17+), and Th1 (CD4+ IFN-*γ*+) in total Th cells were all significantly higher in diabetic patients with CHD (*n* = 42) than those without CHD (*n* = 67).

**Figure 2:**
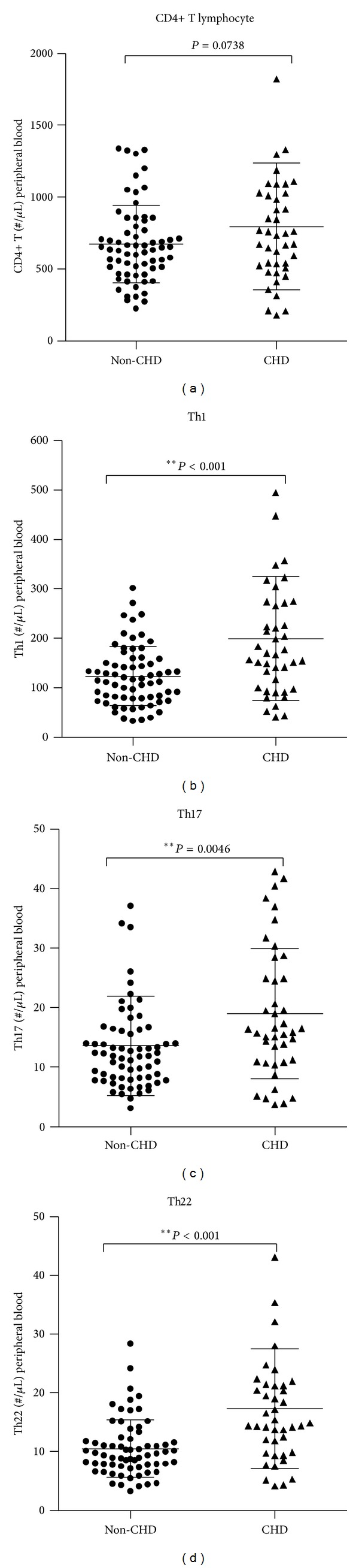
Absolute numbers of Th22, Th1, and Th17 cells were significantly higher in diabetic patients with CHD than those without CHD. Absolute numbers of Th22, Th1, and Th17 cells as well as total CD4+ T cells. (a) Elevating tendency was observed but not statistically significant in absolute numbers of CD4+ T cells in CHD group (*P* = 0.0738). (b) Significant increases in Th1 numbers were observed in CHD group (199.9 ± 125.40) compared to non-CHD group (123.5 ± 59.90) (*P < 0.0001). (c) Significantly elevated percentages of Th17 cells were also found in CHD group (19.01 ± 8.35 versus 13.64 ± 10.94) (*P = 0.0046). (d) Elevations in Th22 cells were more remarkable in CHD group (17.32 ± 10.20 versus 10.53 ± 4.855) (*P < 0.0001). Bars represent SEM.

As suggested by previous works and the demographic characteristics (see Table 1) in our study, the peripheral lymphocyte counts may make a difference in development of CHD. We felt obliged to compare the differences in absolute numbers of specific Th cells in per microliter of peripheral blood between the two groups (shown in Figure 2). Parallel with Th frequencies, the absolute numbers of Th22 together with Th1 and Th17 were significantly higher in diabetic patients with CHD. In fact, though it was not statistically significant, patients with CHD appeared to have higher frequencies of CD4+ T cells ((798.1 ± 440.3 versus 675.8 ± 267.9)/*μ*L, *P* = 0.074).

### 3.3. Proinflammatory Th Cells Expanded in Concert and Promoted the Persistent Inflammation

To further analyze whether Th1, Th17, and Th22 cells expanded in a correlated manner and contributed to the increased inflammatory status, bivariate correlation analysis was performed. Correlation coefficients between each proinflammatory Th subset and CRP were given in Table 2. Here we detected remarkable relationship among Th1, Th17, and Th22, which suggested that elevated proinflammatory Th subsets might act in concert to exacerbate the persisted chronic inflammation thus promoting disease progression. What is more, both absolute numbers and frequencies of Th1 and Th22 turned out to be significantly correlated with plasma CRP levels in our study. Though unspecific, CRP could serve as a good biomarker of inflammation with rough proxy for cardiovascular risk [12], especially when other situations which might cause CRP fluctuation were excluded (see exclusion criteria in Section 2.1). In light of previous studies on molecular basis or animal models, we postulated that these numbers or frequencies of Th subsets represented a proxy for inflammatory context in diabetes and the proinflammatory Th subsets might thus play roles in cardiovascular outcomes in diabetic patients.

### 3.4. Increased Peripheral Proinflammatory Th Subsets Contribute to Cardiovascular Complications in Diabetes

The plausible links between proinflammatory Th subsets and CHD were suggested since we and others have demonstrated their aberrant frequencies or absolute numbers in CHD. However, there was no systemic investigation verifying the independent contribution of specific Th subsets to CHD, especially in diabetes which was characterized by an increase of proinflammatory Th subsets itself. Here we took advantage of binary logistic regression analysis in the case-controlled study to verify the independent contribution of specific Th subsets to CHD onset in diabetic patients. Age, sex, and disease duration of diabetes were considered as confounding variants, and forced entries were arranged in the regression models. As shown in Table 3, our study confirmed that all the frequencies of Th1, Th17, and Th22 contributed to the increased prevalence of CHD. Among them the recently identified Th22 cells exerted the most remarkable impacts. The odds ratio of Th22 frequencies was as high as 41364.708, which indicated that every increase in 1% of Th22 was surprisingly associated with 41364.708-fold increase of risk to get CHD. It was noted that Th22 frequencies distributed in a relative narrow range (1.23%~3.16% in our study) and turned out to be a highly sensitive marker of inflammation (shown in Table 2). Similarly, after being adjusted by age, sex, and duration of diabetes, the absolute numbers of proinflammatory Th cells still represented significant associations with CHD prevalence. The expansion of a single cell in Th1, Th17, and Th22 subset from per microliter of blood was associated with a 1.0%, 5.9%, and 15.9% increased cardiovascular risk, respectively, in diabetic patients. This indicated increased peripheral proinflammatory Th subsets that contributed to development of CHD in diabetes. Higher levels of proinflammatory Th subsets positively correlated with increased cardiovascular complications. And Th22 cells demonstrated the most remarkable effect on CHD development.

To further explore whether the influences of specific Th subsets on CHD were independent of established cardiovascular risk factors, all involved variants including age, sex, disease duration, body mass index, plasma lipid, and CRP levels, with or without history of hypertension, Ln(HOMA-IR), were testified in a stepwise regression model. Plasma levels of CRP, LDL-C, Ln(HOMA-IR), and Th22 number were included in the final model (see Table 4). Th22 cells were proved to be the only independent participant in development of CHD (OR: 1.102; CI: 1.016–1.195; *P* = 0.019) among the proinflammatory Th subsets involved.

### 3.5. Specific Th Subsets Correlated with AIP and Had Potential Predictive Values for CHD in Diabetic Patients

**Figure 3:**
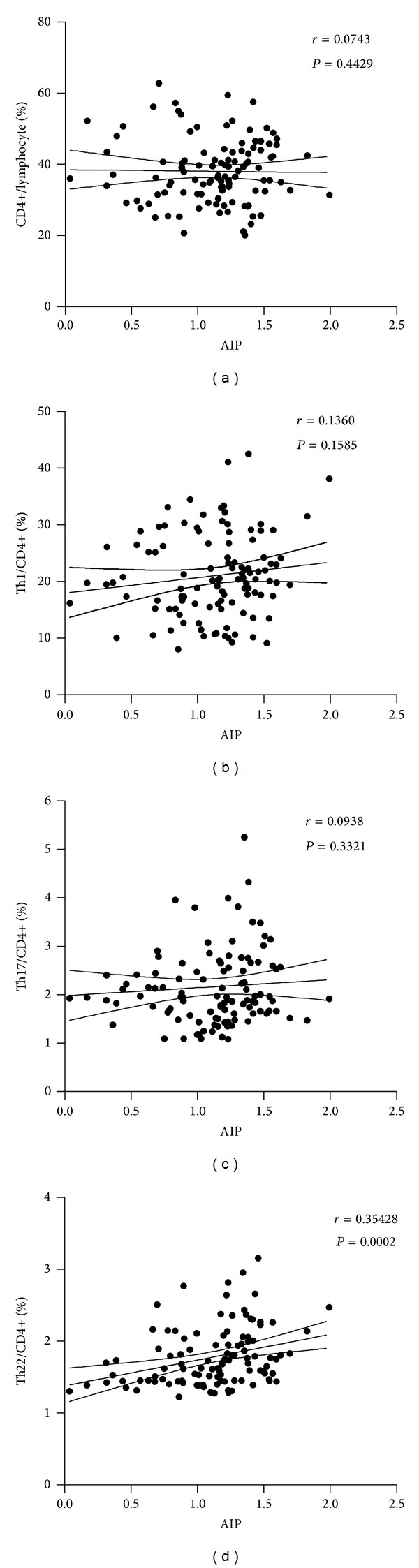
Correlation analysis of peripheral frequencies of Th22, Th1, and Th17 cells with AIP correlation analysis of proinflammatory Th subset frequency with atherogenic index of plasma (AIP) in T2D patients. The positive correlation of Th22 frequency with AIP was proved to be statistically significant (d). However, correlation of AIP with Th1 or Th17 frequency failed to show statistical significance (b, c). Numbers in each figure show Spearman *r* and *P* value, respectively.

**Figure 4:**
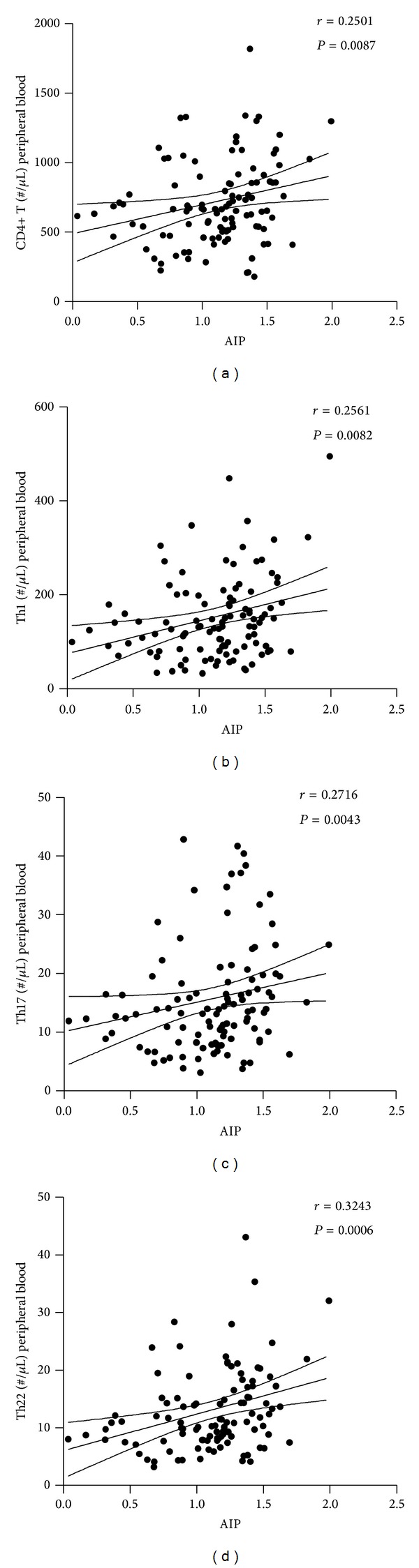
Correlation analysis of absolute numbers of Th22, Th1, and Th17 cells with AIP correlation analysis of the absolute numbers of proinflammatory Th subsets in peripheral blood with AIP in T2D patients. The total number of CD4+ T cells (Th cells) showed a significant positive association with AIP. The positive correlation of Th1, Th17, and Th22 numbers, respectively, with AIP was all proved to be statistically significant (b, c, and d). Numbers in each figure show Spearman *r* and *P* value, respectively.

To evaluate the potential value of aberrant Th subsets in predicating cardiovascular outcomes in diabetes, we first assessed the correlation between the proinflammatory Th subsets and AIP. Non-HDL-C is a well-established atherogenic factor in coronary heart disease, especially in diabetes. And the atherogenic index of plasma could serve as a good predictive index for cardiovascular risk and mortality [13]. Here in our study we revealed significant positive correlations between the absolute numbers of Th22, Th1, and Th17 cells with AIP (see Figure 4). The positive correlation with AIP also proved statistically significant in peripheral frequencies of Th22, but not with total Th cells, Th1 cells, or Th17 cells (see Figure 3). This suggested primarily that both the frequencies of Th22 and the absolute numbers of Th22, Th1, and Th17 cells had potential prognostic values for cardiovascular risk in diabetes. What is more, the frequency or number of Th22 was the most remarkable novel parameter among all involved proinflammatory Th subsets.

Furthermore, categorical analyses in R×C cross-tables were performed to compare the prognostic values of each Th subset and to identify their cutoff values. Generally, the frequencies or numbers of Th cells were categorized into four columns (i.e., normal, mildly elevated, moderately elevated, and severely elevated, resp., as shown in Table 5) by quartile division. The age and sex adjusted odds ratios, 95% confident intervals (CIs), and *P* values were given in Table 5. Again the significantly higher prevalence of CHD was observed in diabetic patients with severe elevation of any proinflammatory subsets. Furthermore, patients with even moderately elevated (higher than the median) Th1 frequency or Th22 number already had a significantly increased risk (4.451- and 67.002-fold, resp.) of CHD. In further analysis in 2 × 2 tables, the frequency of Th1 above the median 20.145% represented an about 5-fold (CI: 2.158–11.82) increased risk of CHD, with a positive predictive value (PPV) of 0.7963 (CI: 0.6647–0.8937) and a negative predictive value (NPV) of 0.5636 (CI: 0.4232–0.6970). And patients with average absolute numbers of Th22 higher than 10.89 per microliter of blood had an about 5-fold (CI: 2.158–11.82) increased risk of CHD, with a sensitivity of 0.6418 (CI: 0.5153–0.7553) and a specificity of 0.7381 (CI: 0.5796–0.8614) (note that the 2 × 2 tables of Th1 frequency and Th22 number, respectively, with CHD prevalence summarized in our study were occasionally the same, thus producing the same statistics). Taken together, our data suggested that the total numbers of Th1, Th17, and especially Th22 cells stood as sensitive proxies for cardiovascular risk.

## 4. Discussion

T2D is accompanied by a 4-fold increase in the incidence of CHD [2]. The impressive correlation between CHD and dysregulation in glucose metabolism has raised the possibility that atherosclerosis and T2D may share common antecedents. The roles of the chronic inflammation in the development of diabetes and its related diseases have been intensively investigated in the first report concerning the elevations of the inflammatory markers in diabetes [14]. Moreover, it has been formulated for half a century that inflammation may be involved in the pathogenesis of atherosclerotic diseases including CHD [15, 16]. In fact, inflammation may rather represent the ultimate common pathway leading to cardiovascular disease. All risk factors such as genetic predisposition [17], aging [18], and psychological stress [19] could lead to cardiovascular disease through the overactivated systemic inflammation. Specific lifestyle patterns, including diet [20], physical activity [21], and smoking [22], also exert much influence on heart disease via their effects on inflammation. Furthermore, it is well illustrated from animal models to molecular mechanisms that the chronic inflammation contributes largely to the peripheral insulin resistance [23]. Thus, the systemic chronic inflammation has been postulated to bridge the increased risk of cardiovascular disease and T2D.

Macrophages were the first well-described inflammatory participant in T2D as well as atherosclerosis, whose recruitment directly contributed to the systemic inflammatory status and plaque formation [24]. More recently, however, it is revealed that an elevation of Th1 and Th17 subsets [25, 26] accompanied by a significant decrease of regulatory T cells (Tregs) [27] may directly trigger the activation and infiltration of macrophage and thus systemic or local inflammation in the development of atherosclerosis and CHD [28]. However, the exact roles and underlying mechanisms of the Th subsets in CHD were far from being elucidated. More importantly, though some of the studies demonstrated an aberrant population of Th subsets in patients, systemic data that could verify the contribution of these changes to CHD were so far absent. To investigate their exact roles in the pathogenesis of CHD in diabetic patients, we examined the frequencies and absolute numbers of peripheral Th cells in patients and made efforts to decode their exact correlations and contributions to the incidence of CHD in diabetes. Moreover, in light of our previous findings concerning the roles of Th22 cells in T2D, we included Th22 cells in our study.

The elevated baseline levels of proinflammatory Th cells in T2D patients have been described previously. In this study, patients with CHD demonstrated impressively higher peripheral frequencies and absolute numbers of Th cells. The aberrant subpopulations of Th cells expanded in a correlated manner and contributed to the persistent inflammation in concert. Furthermore, we verified that increased specific Th subsets contributed to CHD onset in diabetic patients after adjusting for age, sex, and disease duration of diabetes. The impacts of Th1, Th17, and Th22 on the development of CHD were proved to be surprisingly remarkable considering that even one single cell's increase per microliter of blood could result in a significant increase of CHD incidence. To assess whether these associations were independent of the established cardiovascular risk factors, all involved factors including age, sex, disease duration, body mass index, plasma lipid, and CRP levels, with or without history of hypertension, Ln(HOMA-IR), were testified in a stepwise regression model. Only plasma level of CRP and LDL-C, Ln(HOMA-IR), and Th22 number were introduced into the final model. The proinflammatory Th subsets positively correlated with increased cardiovascular complications. The effect of Th22 cells on the development of CHD proved to be most remarkable in our study. It was primarily postulated that elevated Th22 together with Th1 and Th17 contributed to the development of CHD by mediating inflammation and insulin resistance. However, our findings suggested that there were other possible roles of Th22 cells in the development of CHD. Th22 is a recently identified helper T subset with specific phenotype and distinct function from Th1 or Th17 [29]. Th22 has a specific cytokine profile including IL-22 and TNF-*α*, both of which are frequently reported to have important roles in chronic inflammation. IL-22 is bifunctional with both proinflammatory and proliferative effects on tissues depending on the inflammatory context [30]. The impacts of Th22 cells on the development of CHD may be dependent on the synergistic effects of IL-22 and TNF-*α*. In fact, receptors for IL-22 are absent in immune cells but instead restricted to peripheral tissues [30], indicating the possibly direct roles of Th22 to coronary atherosclerosis.

CHD is one of the major complications and comorbidities which accounts for three-quarters of the diabetes-related mortalities [2]. Risk stratification for CHD is thus of great clinical significance in diabetic patients. Previous studies have identified several valuable inflammatory markers as potential indicators of increased CHD risk. Among them the plasma CRP level was the best-described proxy for cardiovascular risks [12]. Our study revealed significant positive correlation between the proinflammatory Th subsets and CRP, which implied the possibility of Th frequency or numbers as new indicators of CHD risk. Similarly, the possible correlation between Th frequency or numbers and the non-HDL-C atherogenic index was evaluated, again demonstrating remarkable associations. Finally, the results of categorical analyses in R×C cross-tables verified the potential prognostic values of proinflammatory Th subsets, especially of Th1 and Th22 cells. In fact, Th1 cells account for a dominating portion in total Th cells and thus contribute a lot to the pro- or anti-inflammatory polarization of total Th plasticity. As suggested in our studies, Th22 cells contributed largely to insulin resistance, which has been identified as a major pathological foundation of atherosclerosis and CHD [24]. Moreover, Th22 might also exert other impacts on the development of CHD independent of the established cardiovascular risk factors. However, the present study has its limitations. Our data only represent a speculative indication of the possibility of Th cells in predicating CHD risk in diabetes. A prospective study must be considered to testify the exact prognostic values of specific Th subsets. Moreover, it remains to be elucidated about the exact roles of Th subsets in CHD patients without T2D. Considering that T2D patients generally have elevated baseline levels of proinflammatory Th cells, the stratification may turn out to be different.

## 5. Clinical Perspectives

- We first identified an even more remarkable increase of Th22 cells alongside Th1 and Th17 in diabetes patients complicated with CHD and revealed its association with the persistent inflammation.
- Our study provided first-hand clinical evidences supporting that systemic chronic inflammation was the central link between the increased risk of cardiovascular disease and T2D while numbers or frequencies of Th1, Th17, and especially Th22 represented a proxy for inflammatory context in diabetes.
- We have testified the adjusted association of specific Th subsets with CHD in a diabetic population by controlling confounding factors like age, sex, duration of illness, and so forth, in the case-controlled study. We evaluated the contribution of each specific Th subset including total Th cells and Th1, Th17, and Th22 cells, respectively, to the incidence of CHD. And the effect of Th22 cells on the development of CHD proved to be the most remarkable in our study.
- Our study suggested that Th22 cells might also play a role in the development of CHD independent of previously established risk factors like insulin resistance, dyslipidemia, and inflammation. The molecular mechanisms remained to be explored. The IL-22-IL-22R1 pathway was suggested.
- The potential prognostic values of specific Th subsets for CHD in T2D were first explored here. Th1 frequency and Th22 numbers showed a predictive value in our study.

## Figures and Tables

**Table 1:** Demographic characteristics of subjects.

	Non-CHD	CHD	*P*
*N*	42	67	
Age (year)	56.79 ± 14.978	61.48 ± 12.73	0.096
Sex (M/F)	19/23	39/28	0.190
Duration (year)	10.10 ± 8.09	11.44 ± 7.50	0.387
BMI (kg/m^2^)	25.00 ± 4.73	25.94 ± 4.72	0.314
Lymphocyte (10^9^/L)	1.76 ± 0.48	2.03 ± 0.89	0.041*
TG (mmol/L)	2.02 ± 1.91	2.05 ± 1.43	0.937
LDL-L (mmol/L)	2.59 ± 0.71	3.35 ± 0.92	0.000**
HDL-L (mmol/L)	1.22 ± 0.28	1.14 ± 0.24	0.134
T-Cho (mmol/L)	4.69 ± 1.01	5.24 ± 1.16	0.010*
CD4+ (%)	37.83 ± 8.30	38.42 ± 10.28	0.743
CRP (mg/L)	2.27 ± 2.34	5.61 ± 4.21	0.000**
ln (HOMA-IR)	0.66 ± 0.81	1.39 ± 0.64	0.000**

BMI: body mass index; non-CHD: patients with type 2 diabettes but without CHD; CHD: patients with type 2 diabetes and CHD; CRP: C-reactive protein; HDL-C: high-density lipoprotein cholesterol; LDL-C: low-density lipoprotein cholesterol; T-Cho: total cholesterol; TG: total triglycerides.

*Statistically significant at level of *P* < 0.05.

**Statistically significant at level of *P* < 0.01.

**Table 2:** Correlation coefficients between each proinflammatory Th subset and CRP.

%	#				
	CRP	Th1	Th17	Th22	CD4+
CRP		0.249**	0.076	0.257**	0.095
		0.009	0.434	0.007	0.325

Th1	0.303**		0.753**	0.740**	0.740**
	0.001		0.000	0.000	0.000

Th17	0.003	0.303**		0.811**	0.816**
	0.972	0.001		0.000	0.000

Th22	0.454**	0.261**	0.811**		0.903**
	0.000	0.006	0.000		0.000

CD4	−0.012	−0.125	−0.030	0.086	
	0.899	0.194	0.761	0.374	

The upper value represents Spearman' rho and the lower corresponding *P *value.

%: correlation between frequencies of corresponding Th subsets/CD4+ Th cells.

#: correlation between absolute counts of corresponding Th cells per milliliter of peripheral blood.

∗: statistically significant at level of *P* < 0.05.

∗∗: statistically significant at level of *P* < 0.01.

**Table 3:** Logistic regression analysis of contribution of specific Th subsets to CHD.

	CD4+/Lym (%)	Th1/CD4+ (%)	Th17/CD4+ (%)	Th22/CD4+ (%)
OR	1.010	1.148**	2.094*	41364.708**
95% CI	0.967–1.055	1.071–1.231	1.184–3.704	535.747–3193746.543
*P*	0.653	0.000	0.011	0.000

	CD4+ (#/*μ*L)	Th1 (#/*μ*L)	Th17 (#/*μ*L)	Th22 (#/*μ*L)

OR	1.001	1.010**	1.059*	1.159**
95% CI	1.000–1.002	1.004–1.015	1.013–1.107	1.072–1.254
*P*	0.080	0.001	0.012	0.000

/Lym (%): percentage in total lymphocytes.

#/*μ*L: absolute counts of corresponding Th cells per milliliter of peripheral blood.

∗: statistically significant at level of *P* < 0.05.

∗∗: statistically significant at level of *P* < 0.01.

**Table 4:** Stepwise logistic regression models for independent risk factors screening.

	*P*	OR	95% CI	
			LL	UL
Step 1a				
CRP	0.000	1.430	1.208	1.693
Constant	0.000	0.176		
Step 2b				
CRP	0.000	1.397	1.173	1.664
HOMA-IR	0.000	3.353	1.760	6.386
Constant	0.000	0.055		
Step 3c				
CRP	0.000	1.401	1.159	1.692
HOMA-IR	0.002	2.976	1.499	5.905
Th22#	0.009	1.104	1.025	1.189
Constant	0.000	0.017		
Step 4d				
CRP	0.002	1.365	1.123	1.660
LDL	0.016	2.310	1.168	4.567
HOMA-IR	0.004	2.827	1.400	5.711
Th22#	0.019	1.102	1.016	1.195
Constant	0.000	0.002		

Th22#: absolute counts of corresponding Th22 cells per milliliter of peripheral blood.

**Table 5:** Categorical analysis of odds ratios adjusted by age and sex.

	Normal	Mildly elevated	Moderately elevated	Severely elevated
CD4+/Lym (%)	OR	0.615	0.824	2.371
	CI	0.185–2.044	0.257–2.636	0.784–7.173
	*P*	0.427	0.744	0.126
Th1/CD4+ (%)	OR	2.077	5.451	9.503
	CI	0.523–8.241	1.456–20.413	2.500–36.116
	*P*	0.299	0.012*	0.001**
Th17/CD4+ (%)	OR	0.649	3.000	4.584
	CI	0.167–2.517	0.910–9.892	1.402–14.983
	*P*	0.532	0.071	0.012*
Th22/CD4+ (%)	OR	0.713	2.667	7.125
	CI	0.178–2.858	0.771–9.220	2.081–24.398
	*P*	0.633	0.121	0.002**

CD4+ (#/*μ*L)	OR	0.533	1.174	1.196
	CI	0.164–1.735	0.389–3.544	0.397–3.598
	*P*	0.296	0.776	0.075
Th1 (#/*μ*L)	OR	0.942	2.543	5.717
	CI	0.254–3.489	0.758–8.534	1.709–19.122
	*P*	0.928	0.131	0.005**
Th17 (#/*μ*L)	OR	1.528	1.665	3.900
	CI	0.454–5.145	0.513–5.400	1.217–12.495
	*P*	0.493	0.396	0.022*
Th22 (#/*μ*L)	OR	3.113	68.002	112.9
	CI	0.121–79.94	3.771–1233	6.192–2058
	*P*	1.000	0.000**	0.000**

/Lym (%): percentage in total lymphocytes.

#/*μ*L: absolute counts of corresponding Th cells per milliliter of peripheral blood.

∗: statistically significant at level of *P* < 0.05.

∗∗: statistically significant at level of *P* < 0.01.
